# Validity and reliability study of a novel surface electromyography sensor using a well-consolidated electromyography system in individuals with cervical spinal cord injury

**DOI:** 10.1038/s41393-024-00981-y

**Published:** 2024-04-04

**Authors:** Chandrasekaran Jayaraman, Chaithanya Krishna Mummidisetty, Arun Jayaraman, Kimberly Pfleeger, Michelle Jacobson, Melissa Ceruolo, Ellora Sen-Gupta, James Caccese, David Chen

**Affiliations:** 1 Max Näder Center for Rehabilitation Technologies and Outcomes Research, Shirley Ryan AbilityLab, Chicago, IL USA; 2 AbbVie Inc, North Chicago, IL USA; 3 Medidata Solutions, a Dassault Systèmes company, New York, NY USA

**Keywords:** Trauma, Spinal cord diseases

## Abstract

**Study design:**

Non-interventional, cross-sectional pilot study.

**Objectives:**

To establish the validity and reliability of the BioStamp nPoint biosensor (Medidata Solutions, New York, NY, USA [formerly MC10, Inc.]) for measuring electromyography in individuals with cervical spinal cord injury (SCI) by comparing the surface electromyography (sEMG) metrics with the Trigno wireless electromyography system (Delsys, Natick, MA, USA).

**Setting:**

Participants were recruited from the Shirley Ryan AbilityLab registry.

**Methods:**

Individuals aged 18–70 years with cervical SCI were evaluated with the two biosensors to capture activity on upper-extremity muscles during two study sessions conducted over 2 days (day 1–consent alone; day 2–two data collections in same session). Time and frequency metrics were captured, and signal-to-noise ratio was determined for each muscle group. Test-retest reliability was determined using Pearson’s correlation. Validation of the BioStamp nPoint system was based on Bland-Altmann analysis.

**Results:**

Among the 11 participants, 30.8% had subacute cervical injury at C5–C6; 53.8% were injured within 1 year of the study. Results from the test-retest reliability assessment revealed that most Pearson’s correlations between the two sensory measurements were strong (≥0.50). The Bland-Altman analysis found values of the signal-to-noise ratio, frequency, and peak amplitude were within the level of agreement. Signal-to-noise ratios ranged from 7.06 to 22.1.

**Conclusions:**

In most instances, the performance of the BioStamp nPoint sensors was moderately to strongly correlated with that of the Trigno sensors in all muscle groups tested. The BioStamp nPoint system is a valid and reliable approach to assess sEMG measures in individuals with cervical SCI.

**Sponsorship:**

The present study was supported by AbbVie Inc.

## Introduction

Traumatic cervical spinal cord injury (SCI) is a devastating event that results in wide-ranging functional limitations and abilities that change with time post-injury [1–3]. Although the upper limb strength measurement component of the International Standards for Neurological Classification of Spinal Cord Injury (ISNCSCI) is the primary subjective evaluation tool used in clinical research to examine upper extremity neurological recovery, the upper limb tasks are complex and often asymmetrical, and strength measures are often not enough to capture all changes that happen at the neuromuscular level post-SCI [4, 5]. Additional validated tools designed to assess clinically meaningful function at the neuromuscular level are needed to provide information on the natural recovery process and the potential benefit of a drug or other therapeutic interventions during clinical trials [4].

Biosensor-based approaches that quantify surface electromyography (sEMG) signals may be more sensitive than standard clinical assessments, like manual muscle tests (MMT), in detecting neuromuscular function. One limitation of MMT is the subjective nature of testing and variability between assessors [6]. For example, MMTs have a “ceiling effect,” which rates the best strength as a 5, even in the presence of functional strength deficits; “floor effect” occurs when the assessor rates strength as a 0, although EMG may detect a signal [6]. sEMG assessments are not limited by ceiling effects [1]. This enhanced sensitivity of sEMG assessments allows for earlier and more sensitive detection of neuromuscular recovery when signals are minimal and sporadic, which is difficult to quantify using standard muscle function tests alone [7, 8].

Most existing sEMG systems that can measure subclinical muscle activation require technical expertise and technologically complex set-up strategies that may not be available at all clinical sites (e.g. the Delsys Trigno™ wireless electromyography system [Delsys, Natick, MA, USA] is laptop rather than cloud-based) [5, 7, 8]. Wearable sEMG biosensors are attached to the skin using a disposable adhesive and are integrated with cloud-based data management systems. The sensors are streamlined and can automate the measurement of muscle activity, thereby playing a vital role in acute-to-chronic SCI investigational drug trials by minimizing the burden placed on the study participant and clinical research staff and supplement clinical assessments [9]. When implemented correctly, cloud-integrated wearable sEMG biosensors are beneficial in the context of multi-site clinical trials in minimizing assessment variability that can result from individual participant characteristics, sensor placement, data collection, and sensor parameters [5].

The purpose of this pilot study was to establish the validity and reliability of sEMG outcome metrics derived from a novel cloud-based, wireless, flexible, and wearable biosensor (BioStamp nPoint [Medidata, New York, USA]) by comparing the sEMG outcome metrics with the gold standard reference for sensitive sEMG measurements. Furthermore, the study establishes optimal sEMG sensor placement and signal quality for reliable measurement of motor impairment in individuals with cervical SCI at both the early recovery and chronic stage.

## Methods

### Study design and participants

Individuals with cervical SCI were evaluated with two biosensors to capture muscle activity in a non-interventional and cross-sectional pilot study. Participants were recruited from a research registry maintained by the Shirley Ryan AbilityLab (Chicago, IL, USA) and evaluated between August 2020 and December 2020. Based on a power of 0.8 (beta = 20%), alpha = 0.05 (two tailed), effect size 0.6 and standard deviation of 0.6, the estimated sample size for a pairwise test was *n* = 10. Accounting for 20% attrition, *n* = 11 was sufficient for the pairwise signal comparison. The study protocol was approved by the Northwestern University (Evanston, IL, USA) institutional review board before study initiation and each participant provided written informed consent.

Eligible participants were aged 18–70 years and had experienced a traumatic cervical SCI event (neurological level of injury, C4–C7) with no evidence of complete cord transection 1 week to 2 years before enrollment (confirmed in electronic medical records). Participants were able to tolerate sensors on the skin surface and were not misusing drugs or alcohol or using tobacco products. Study exclusion criteria were unstable neurological and/or cardiovascular symptoms, uncontrolled hypertension and/or diabetes mellitus, chronic obstructive pulmonary disease, emphysema, severe asthma, cancer, and/or other comorbidities. Participants currently taking a selective serotonin reuptake inhibitor or tricyclic antidepressant, who had received a BOTOX^®^ injection within the last 3 months, or had a pacemaker or antispasticity implantable pump were ineligible to enroll in the trial.

### Materials

#### Electrodes

The electrodes utilized in this study were circular, 10 mm, carbon doped ABS coated with silver/silver chloride.

#### Biosensors

The BioStamp nPoint system (Medidata, New York, USA) is a US Food and Drug Administration 510 (k) class-II medical device cloud-based platform designed to collect medical-grade physiological data. The BioStamp nPoint sensor collects raw data including sEMG, acceleration, and gyroscope signals. The sensors are multi-modal, multi-location, rechargeable, and reusable. An investigator-facing tablet application allows for sensor assignment and viewing of streamed data during data collection. Researchers and clinicians designed, configured, and managed data collection via the BioStamp nPoint investigator web portal. Additional BioStamp nPoint system specifications are shown in Supplementary Table 1.

#### Gold standard used

The Delsys Trigno^TM^ wireless electromyography system (Delsys) is currently the gold standard for sEMG signal detection [10]. Each sensor has an electromyography electrode and three-axis accelerometer that can transmit data wirelessly within a range of 40 m and the rechargeable battery lasts for 7 h. Data acquisition was managed using Delsys proprietary EMGworks Acquisition software. Additional Delsys Trigno wireless electromyography system specifications are shown in Supplementary Table 2. The differences between systems are outlined in Supplementary Table 3.

### Assessments

**Fig. 1:**
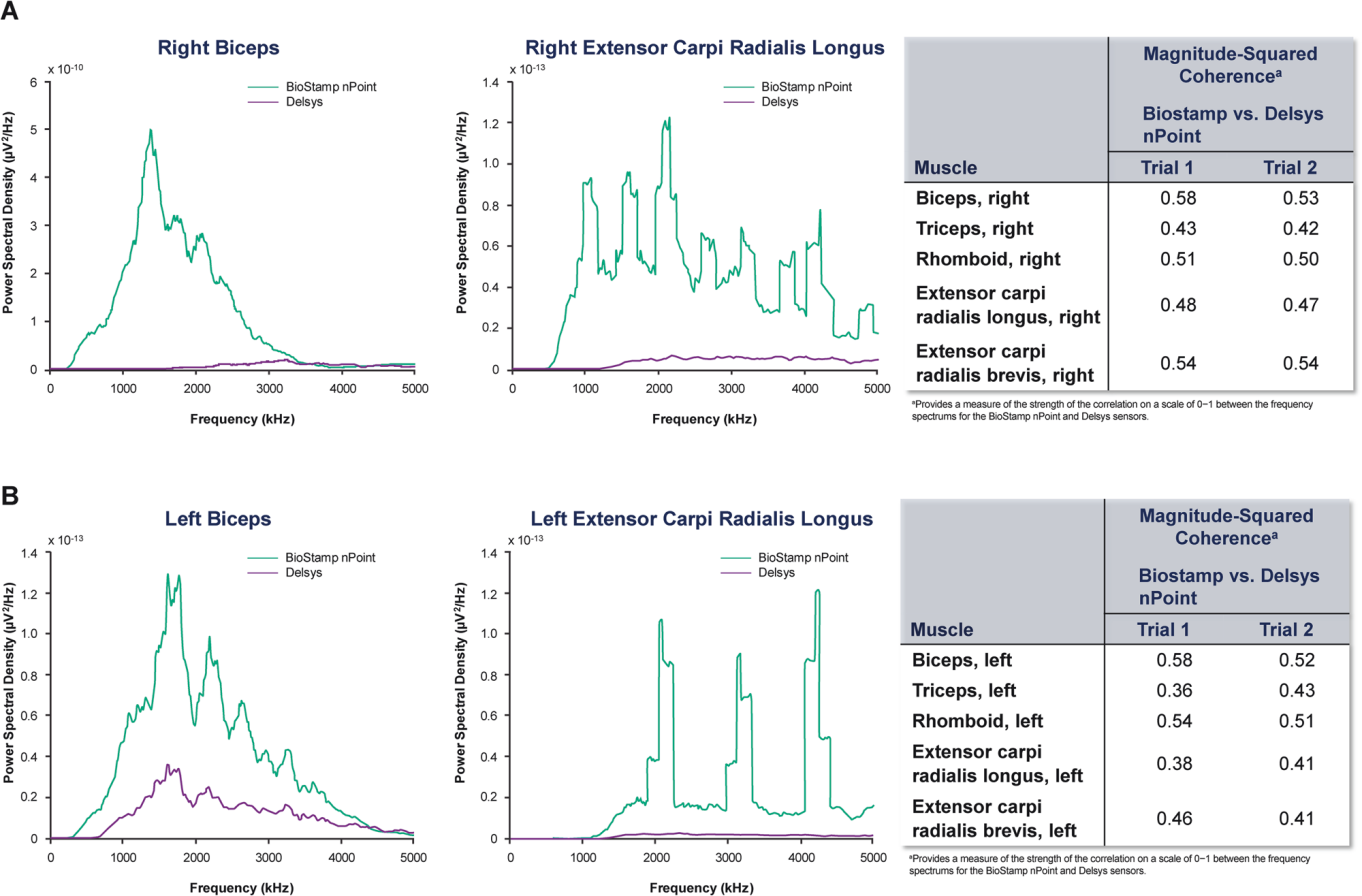
Comparison of frequency metrics between the Delsys and BioStamp nPoint systems. The power spectral density (PSD) and coherence comparisons for each trial are shown. Coherence >0.5 implies high correlation. *BioStamp nPoint* BioStamp nPoint electromyography system; *Delsys* Delsys Trigno wireless electromyography system. **A** Right biceps, right extensor carpi radialis longus; **B** left biceps, left extensor carpi radialis longus.

Study participation included two study sessions that lasted up to 3 h each and were conducted over 2 days (participant consented on day 1 and sensors were tested on two different instances on day 2). Biosensors (BioStamp nPoint and Delsys) were placed on eight upper-extremity muscles (biceps [C4–C5], triceps [C7], rhomboids [C5], and extensor carpi radialis longus and brevis [C6–C7]). The location of muscles was identified by palpation. Each participant’s skin surface was carefully cleaned with alcohol wipes. The electrodes were affixed to the skin with hypoallergenic tape to reduce movement artifacts. Each electrode placement location was based on anatomical landmarks described in Supplementary Table 4 [11]. Movement information was collected from the accelerometer and/or gyroscope in addition to the sEMG data. Standard skin preparation techniques to clean the skin were used before placement of the biosensors, and adhesive tapes/elastic bandages were used to secure the sEMG sensors to the skin surface. MMTs were performed by experienced physical therapists to evaluate muscle strength and activity. Electromyography procedures were explained to participants before the start of testing. The muscle was isolated and gradual external force was applied at a 90-degree angle to the long axis of the muscle. Each muscle was scored on a graded scale of 1 (“weak”) to 5 (“strong”) based on the participant’s external force resistance. Participants could be tested while prone to eliminate the effect of gravity. Participants were subjected to six MMTs (two trials of three contractions/muscle) for each muscle group with a rest break of 10–12 min between tests to minimize fatigue. Data analysis or post-processing of the EMG data was conducted using MATLAB 2020b/2021 (Mathworks, Natick, MA, USA). A 12th order bandpass Butterworth filter with edge frequencies of 0.02π and 0.8π radians/sample was used to filter the electromyography data. Standard EMG post-processing methods described in rehabilitation research literature were used [8]. For frequency spectrum, a Fast Fourier transform was applied to input signal. When the input was not pure unmodulated sine wave, the harmonics of the fundamental frequency appeared periodically at higher frequencies, as seen in Fig. 1. Due to differences in the amplification gain factor in the hardware of both EMG units, a magnitude-based comparison is not a reasonable metric. To establish equivalence of frequency spectra, we used a metric called Coherence (equivalent of correlation in frequency domain; a standard practice in signal processing). A rectification was implemented on the filtered raw EMG [12].

### sEMG sensor-derived outcomes

During each visit, the recorded sEMGs from the BioStamp nPoint and Delsys systems were assessed. To investigate sEMG signal quality, the time and frequency domain metrics were captured with both systems [13]. The time-domain metrics analyzed included signal-to-noise (decibel) ratio (SNR), peak amplitude (millivolt), burst duration (millisecond), and burst variance [13]. Methods for the application of EMG recording published by Wu et al were utilized [12]. The frequency domain metrics collected were the mean, median, peak frequency, and spectrum. The SNR was determined for each muscle group with the BioStamp nPoint system to evaluate the electromyography signal characteristics at each location. The assessments were repeated for each muscle group in two separate trials to determine test-retest reliability correlations.

### Data analysis

Post-processing of data from both Delsys and BioStamp nPoint systems were completed using MATLAB 2021 (MathWorks Inc). Standard electromyography post-processing methods obtained from rehabilitation literature were used (see McManus et al.) [8]. The Delsys system’s results were used for comparisons and for validating data from the BioStamp nPoint system.

### Statistical analysis

A Bland-Altman and correlation statistical analysis with 95% confidence interval was performed between the BioStamp nPoint sEMG and the Delsys Trigno gold standard sEMG to establish statistical validity. Based on 95% or above confidence interval criteria, the optimal biosensor parameters were recommended. The time and frequency domain metrics from the sensor output were compared to ascertain reliability and validity. Additionally, we used advanced pattern classification and machine learning algorithms to data mine outcomes from the post-processed sensor data to correlate it with the assessed MMT scores by therapists. The analysis provided information on the amount of agreement between human vs sensor-based assessment/measurement.

Pearson correlation analysis (threshold of low <0.3, medium 0.3–0.49, or high ≥0.5) was used for test-retest reliability of the sEMG signal time and frequency domain of the BioStamp nPoint compared with the Delsys Trigno wireless sEMG sensors. Test-retest reliability of the sEMG outputs between trial 1 and trial 2 (of the same sensor) were also compared using a Pearson’s correlation coefficient (insubstantial <0.1, low 0.1–0.29, moderate 0.3 to 0.49, and high ≥0.5). To compare optimal sensor placement, the signals between BioStamp nPoint and Delsys Trigno wireless sensors were compared using Spearman correlation because data were not normally distributed (insubstantial <0.1, low 0.1–0.29, moderate 0.3–0.49, and high ≥0.5).

### Cloud-based storage of sEMG data

All recorded and processed data collected from the BioStamp nPoint device were stored in a secure cloud. The sensor-recorded data were transferred between components using secure protocols and encryption. Data were collected by the on-skin sensors and stored on internal servers. Once the wear cycle was completed and the sensors were placed on the dock, the data were transferred to and temporarily stored in a smart dock secure digital memory card. After the smartphone was docked, data were moved to the cloud via a secure Hypertext Transfer Protocol application.

When the smartphone and dock received confirmation that the BioStamp nPoint cloud had acquired data, the data were cleared from the kit. The data in BioStamp nPoint cloud are accessible via an application programming interface and for authorized operators of the system through the web application interface. Additionally, data in transit are encrypted between firmware to mobile device and mobile device to cloud.

## Results

### Demographics and baseline characteristics

In this study, 15 individuals with traumatic cervical SCIs were screened. Of the 13 that qualified for participation, 11 completed the study with one participant being removed due to scheduling difficulties. Ten participants (83.3%) were male, and most were White (66.7%). The mean (SD) age was 48 (17.9) years (Table 1). More than half (53.8%) of the participants experienced acute cervical SCI within the last year, and 30.8% of participants had a cervical injury at C5–C6. In most muscle groups tested, the mean MMT grade was above 4; mean grades for the left and right rhomboid muscle groups were 3.7 (Table 1).

**Table 1:** Baseline demographics and characteristics.

Parameters	Total, n (%) (*N* = 11)	
Demographics		
Age (years), mean (SD)	48 (17.9)	
Male, %	83.3	
Race, White %	66.7	
Injury level		
C4	1 (7.7)	
C4–C6	1 (7.7)	
C4–C7	1 (7.7)	
C5	1 (7.7)	
C5–C6	4 (30.8)	
C5–C7	1 (7.7)	
C6	1 (7.7)	
C6–C7	1 (7.7)	
Chronicity		
Acute, for the duration of <1 year	7 (53.8)	
Acute, for the duration of 1 year	1 (7.7)	
Chronic, for the duration of >1 and <2 years	3 (23.1)	
Chronic, for the duration of 2 years	1 (7.7)	

Manual Muscle Test score^a^	Mean (SD)^b^	Median (Min, Max)^b^
Biceps, left	4.83 (0.38)	5 (4, 5)
Biceps, right	4.58 (0.78)	5 (3, 5)
Triceps, left	4.21 (0.88)	4 (2, 5)
Triceps, right	4.04 (1.04)	4 (2, 5)
Rhomboid, left	3.72 (0.57)	4 (2, 4)
Rhomboid, right	3.68 (0.67)	4 (2, 4)
Extensor carpi radialis longus, left	4.55 (0.91)	5 (2, 5)
Extensor carpi radialis longus, right	4.41 (1.01)	5 (2, 5)
Extensor carpi radialis brevis, left	4.57 (0.93)	5 (2, 5)
Extensor carpi radialis brevis, right	4.41 (1.01)	5 (2, 5)

*C* Cervical, *max* maximum, *min* minimum.

^a^Manual Muscle Test score range 0–5 (0 lowest, 5 highest).

^b^Data are presented as mean (SD) or median (min, max) of all sessions (trials 1 and 2 for all participants).

### sEMG sensor-derived outcomes

Across the four upper-extremity muscles tested, the frequency domain metrics were in agreement with the BioStamp nPoint and Delsys sensor types bilaterally (Table 2). Among the time-domain metrics, most were similar between the two systems, except in most cases peak amplitude measured higher in the BioStamp vs Delsys sensors bilaterally.

**Table 2:** BioStamp nPoint vs Delsys Systems: time and frequency metrics.

Metrics^a^	Biceps (C4–C5)		Triceps (C7)		Rhomboids (C5)		Extensor Carpi Radialis Longus and Brevis (C6–C7)	
	BioStamp nPoint	Delsys	BioStamp nPoint	Delsys	BioStamp nPoint	Delsys	BioStamp nPoint	Delsys
Time Domain								
Signal-to-noise (dB) ratio	R: 22.1 (7.9) L: 19.6 (7.1)	R: 24.6 (9.7) L: 23.0 (8.1)	R: 12.4 (6.4) L: 11.0 (5.7)	R: 14.7 (9.5) L: 17.7 (10.8)	R: 10.0 (6.5) L: 8.2 (6.6)	R: 12.3 (7.5) L: 11.3 (9.3)	R: 14.3 (6.9) L: 14.7 (8.1)	R: 24.3 (6.3) L: 25.5 (9.1)
Peak amplitude (mV)	R: 0.0036 (0.0026) L: 0.0026 (0.0015)	R: 0.0013 (0.0013) L: 0.0011 (0.0007)	R: 0.001 (0.001) L: 0.0008 (0.0009)	R: 0.0003 (0.0005) L: 0.0003 (0.0003)	R: 0.0013 (0.0012) L: 0.001 (0.001)	R: 0.0004 (0.0003) L: 0.0005 (0.0005)	R: 0.001 (0.001) L: 0.001 (0.001)	R: 0.0011 (0.0009) L: 0.0013 (0.0015)
Burst Time (mS)	R: 5.9 (1.2) L: 5.3 (0.9)	R: 6.3 (1.1) L: 5.9 (1.1)	R: 6.3 (1.8) L: 6.9 (3.3)	R: 6.8 (1.6) L: 6.3 (1.9)	R: 9.0 (2.6) L: 8.6 (1.8)	R: 9.5 (2.6) L: 8.2 (2.0)	R: 5.7 (1.2) L: 5.9 (1.6)	R: 5.8 (1.2) L: 6.3 (1.6)
Burst variance	R: 7.2 e^−7^ (8.9 e^−7^) L: 3.6 e^−7^ (4.5 e^−7^)	R: 1.0 e^−7^ (1.9 e^−7^) L: 4.9 e^−8^ (4.8 e^−8^)	R: 1.2 e^−7^ (2.3 e^−7^) L: 8.6 e^−8^ (1.8 e^−7^)	R: 1.8 e^−8^ (4.1 e^−8^) L: 9.8 e^−9^ (1.4 e^−8^)	R: 1.3 e^−7^ (1.5 e^−7^) L: 1.0 e^−7^ (1.1 e^−7^)	R: 1.2 e^−8^ (2.2 e^−8^) L: 2.0 e^−8^ (3.3 e^−8^)	R: 1.4 e^−7^ (2.0 e^−7^) L: 1.6 e^−7^ (2.1 e^−7^)	R: 5.4 e^−8^ (6.1 e^−8^) L: 1.6 e^−7^ (3.7 e^−7^)
Frequency Domain								
Frequency	R: 60.9 (7.8) L: 62.3 (6.4)	R: 82.8 (18.6) L: 80.6 (9.3)	R: 66.3 (13.1) L: 71.2 (14.3)	R: 79.9 (8.7) L: 82.8 (12.6)	R: 61.5 (13.8) L: 51.5 (9.8)	R: 67.7 (11.1) L: 71.8 (13.9)	R: 87.8 (12.5) L: 80.9 (15.2)	R: 89.8 (17.9) L: 100.3 (23.9)
Peak Frequency	R: 49.7 (7.5) L: 47.0 (6.7)	R: 56.3 (10.7) L: 55.4 (8.3)	R: 42.6 (11.7) L: 43.4 (15.3)	R: 58.5 (36.2) L: 48.9 (8.2)	R: 43.5 (15.0) L: 36.7 (13.9)	R: 43.9 (6.0) L: 44.1 (9.9)	R: 53.6 (12.3) L: 48.7 (14.3)	R: 54.3 (11.0) L: 61.7 (20.9)

^a^Data are mean (SD), unless otherwise specified.

*BioStamp nPoint* BioStamp nPoint electromyography system*, C* Cervical, *Delsys* Delsys Trigno wireless electromyography system, *dB* decibel, *mS* millisecond, *mV* millivolt.

### BioStamp nPoint system reliability

**Fig. 2:**
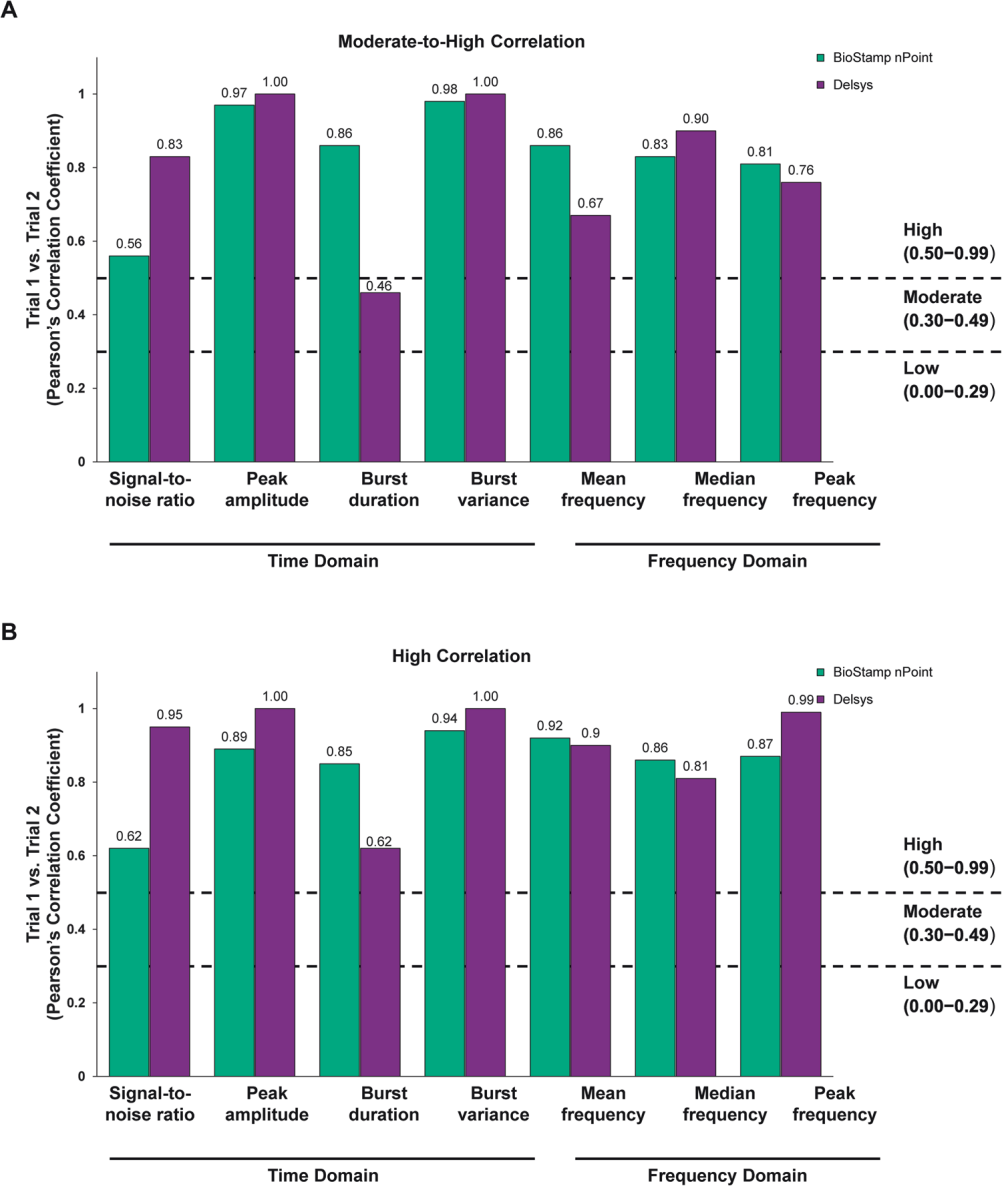
Test-retest reliability Pearson correlation between measurements: triceps time and frequency metrics. Data are from measurements obtained from the triceps bilaterally (*n* = 11). **A** right triceps, **B** left triceps.

Two trials were performed to assess the reproducibility or test-retest reliability correlation of BioStamp nPoint system data capture. Overall, most Pearson correlations were strong (≥0.50) in the time and frequency domain parameters for the four upper-extremity muscle groups (Supplementary Tables 5, 6). Figure 2 shows that the time and frequency domain correlations for both triceps muscle were strong (≥0.50) except for the burst duration time domain, which was moderate (0.46) on the right side. Supplementary Tables 5, 6 demonstrate similar moderate (0.30 to ≤0.49) to strong (≥0.50) correlations in time domain and frequency domain parameters across the two trials for the other upper-extremity muscle groups, with the exception of peak frequency in the extensor carpi radialis longus bilaterally, with a low correlation (<0.30).

### BioStamp nPoint system validation

**Fig. 3:**
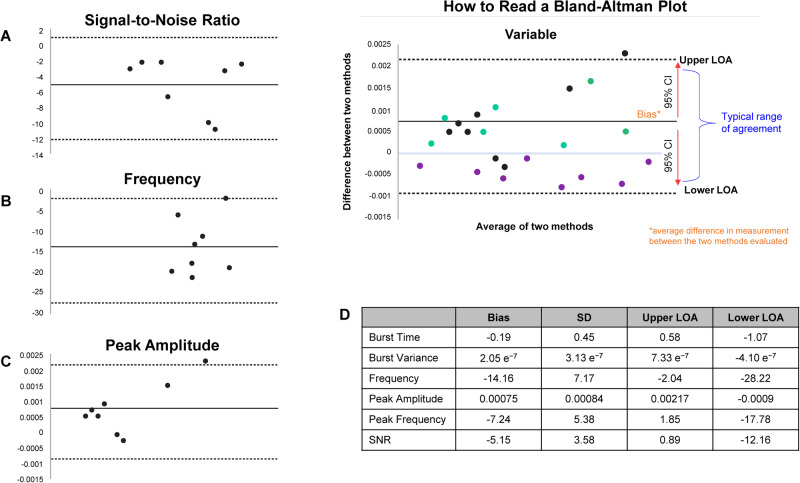
Bland-Altman plots showing the degree of correlation. Bland-Altman of **A** sEMG SNR, **B** frequency, **C** peak amplitude properties between the BioStamp nPoint electromyography and Delsys Trigno^TM^ system, and **D** summary findings for all domains. The callout box shows a sample Bland-Altman plot to aid in data interpretation. In the sample plot, the scatter of black data points above and below the 0 line indicates no bias in favor of one method over the other with respect to the dimension measured. Datasets clustering above or below the zero line, as shown in green and purple, indicate that one method tends to overestimate or underestimate values [21]. CI Confidence interval, LOA Level of agreement, SD Standard deviation, SNR Signal-to-noise ratio. Note that 8 dots correspond to the 8 muscles tested.

Figure 3 presents Bland-Altman plots for select time and frequency domain parameters and summary data for all key metrics for the eight muscles tested, demonstrating values for the signal-to-noise ratio, frequency, and peak amplitude fell within the limits of agreement.

### Optimal sampling and sensor placement

**Fig. 4:**
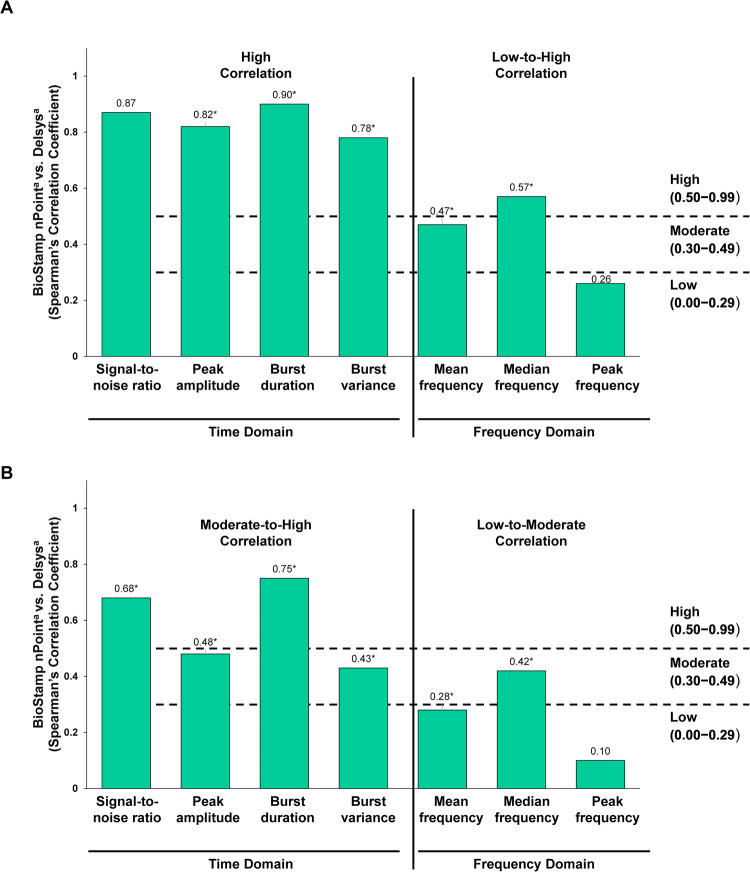
BioStamp nPoint vs Delsys: biceps time and frequency metrics. Data are measurements obtained from the biceps. Statistical significance comparing the signal mean values was determined using the non-parametric, Wilcoxon signed-rank test. **A** Right biceps, **B** left biceps. *P < 0.05. Some data were lost due to poor signal quality. ^a^*n* = 11. *BioStamp nPoint* BioStamp nPoint electromyography system, *Delsys* Delsys Trigno wireless electromyography system.

Figure 4 shows the Spearman correlation coefficient analysis, to assess for optimal sampling and sensor placement, of the time domain and frequency domain results for the biceps muscle. Bilateral biceps had moderate (0.30 to ≤0.49) to strong (≥0.50) correlations in the time domain parameters and weak (<0.30) to strong (≥0.50) correlations for the frequency domain metrics.

Spearman’s correlation coefficient analysis of the time domain metrics from the BioStamp nPoint and Delsys sensors revealed that most correlations were strong (≥0.50) in the biceps, triceps, and rhomboid muscles (Supplementary Table 7). The time domain results from the extensor carpi radialis longus muscles had weak correlations (<0.30) between the two sensor types, except the for burst duration time domain (Supplementary Table 7). The correlations between the two sensors for the frequency domain parameters were primarily weak (<0.30) to moderate (0.30–0.49) in the upper-extremity evaluations (Supplementary Table 8). Figure 1 shows that the coherence between the two sensors are moderate-to-high range for all muscle groups (≥0.3). This implies that both signals have moderate-good linear correlation in the Fourier spectrum as well. The amplitude differences in the frequency spectrum between the two biosensors arise from the gain factor due to inherent proprietary hardware differences (circular disc electrode for the BioStamp nPoint and rectangular-shaped electrode for the Delsys system). Therefore, it is recommended that direct amplitude comparison between the two biosensors be avoided.

The SNR captured in this study ranged from 7.1 to 22.1, which is sufficient to capture sEMG data with the BioStamp nPoint sensors (Supplementary Table 9). The highest SNR was observed in the biceps, which was approximately twofold higher than the triceps, rhomboid, and extensor carpi radialis longus muscles. The optimal sampling rate for measurement was 1000 Hz, the maximum option for the electrodes in the BioStamp nPoint sensors and a rate that was able to be shared between the two systems. Data from the Delsys system sensors was downsampled to 1000 Hz for comparison.

## Discussion

The BioStamp nPoint system performed similarly to the gold standard Delsys wireless electromyography system for the MMT tasks studied in this cohort. The time and frequency domain results were similar between the BioStamp nPoint and the Delsys sensor types across the upper-extremity muscle groups in individuals with cervical SCI, despite the differences in the frequency spectrum between the two systems. Results from the test-retest reliability analyses and Pearson’s correlations demonstrate the reliability of the BioStamp nPoint system, while results from the Bland-Altman analyses confirm the BioStamp nPoint system is a valid approach to detect sEMG and measure muscle activity.

Muscle strength and recovery are both highly correlated with sEMG amplitude [14, 15], and sEMG can serve as an indicator of muscle health and function [16]. Sensors can detect muscle activity in the absence of visible movement, while at rest or under passive movement, and in muscles where strength measurement is challenging [1]. Sensors can also be used to assess complex motor tasks by measuring the activity of multiple muscles simultaneously, and can be used to detect reflex activity inherent in the spinal cord [1, 17]. Characterizing sEMG in the context of SCI is important in understanding the natural recovery process and the potential efficacy of a drug or other intervention.

When implemented correctly, sensor-based approaches are especially beneficial in the context of multisite clinical trials as they can aid in minimizing the variability and bias introduced by more subjective methods of assessment, such as MMT [6]. However, the reliability of sEMG measurements is directly governed by parameters such as the number of sensors, sensor placements (location), data sampling settings, and sensor cycle times [5, 18]. It is, therefore, critical to determine the optimal digital biosensor parameters and procedures to standardize sensor administration, data collection, and data handling for the BioStamp nPoint system.

The optimal sensor placement of the BioStamp nPoint sensors will aid and standardize protocols across multiple clinical trial site evaluations participating in the phase 2 multi-site clinical trial for specific drugs. Sensor placement may need to be modified depending on the individual’s condition, bracing, or postural requirements [11]. To produce reliable measurements and minimize human error, clinical trials should establish a strategy to handle unexpected changes in sensor placement [7]. Based on the results from this study, a recommendation manual for the phase 2 clinical trial will be created for optimal BioStamp nPoint system parameters and sensor placement.

Wearable sEMG biosensors can capture muscle activity at the neuromuscular level and supplement clinical assessments, although a few considerations should be taken into account when using sEMG biosensors. The individual’s current use of selective serotonin reuptake inhibitors or a history of BOTOX injections and/or antispasmodic therapy, which can impact muscle firing and/or confound the sEMG outcomes, should be considered before performing sEMG assessments [19]. The upper-extremity assessments were static tasks and the results from this study may generalize directly to dynamic tasks, such as walking. However, it is worth noting that the BioStamp nPoint system has been previously validated in individuals with multiple sclerosis to analyze balance impairment and sway metrics and other disease states [20].

There were several limitations of this study. The phase 2a clinical trial will explore sEMG collection at several times during the study. The pilot study included test-retest reliability but always on the same day. It will be important to determine the effect of testing with weeks between assessments. Although regaining hand function is a top priority in treating patients who have a cervical SCI, finger flexors and thenar muscles were not a feasible choice for electrode placement, given the larger footprint of the BioStamp nPoint sensors. Therefore, larger muscle groups in the upper arm and forearm were used to establish the validity and reliability of the sensors. The root-mean-square of the EMG signal was not analyzed and reported because we were more interested in the signal-to-noise characteristics. In addition, there is a limitation of a maximum 1000 Hz threshold for the BioStamp nPoint sensors.

Key advantages to using the BioStamp nPoint system include its wireless, flexible features, and the utilization of a cloud-based platform that is secure and scalable across a multi-center study. The seamless collection, transfer, review, and export of data will facilitate the standardization of sEMG data analyses across different trials and study sites. The validated and reliable BioStamp nPoint system will be used in the phase 2 clinical trial (NCT04295538) to assess muscle activity in individuals with cervical SCI.

### Supplementary information

Supplemental Material

Reporting Checklist Summary

## Data Availability

AbbVie is committed to responsible data sharing regarding the clinical trials we sponsor. This includes access to anonymized individual and trial-level data (analysis data sets), as well as other information (eg, protocols, clinical study reports, or analysis plans), as long as the trials are not part of an ongoing or planned regulatory submission. This includes requests for clinical trial data for unlicensed products and indications. These clinical trial data can be requested by any qualified researchers who engage in rigorous, independent, scientific research, and will be provided following review and approval of a research proposal, Statistical Analysis Plan (SAP), and execution of a Data Sharing Agreement (DSA). Data requests can be submitted at any time after approval in the US and Europe and after acceptance of this manuscript for publication. The data will be accessible for 12 months, with possible extensions considered. For more information on the process or to submit a request, visit the following link: https://vivli.org/ourmember/abbvie/ then select “Home”.
