# Supplementary material for: Validity and reliability study of a novel surface electromyography sensor using a well-consolidated electromyography system in individuals with cervical spinal cord injury
Source: Spinal Cord. 2024 Apr 4;62(6):320–7. doi: 10.1038/s41393-024-00981-y (PMC11199136; doi:10.1038/s41393-024-00981-y)
Supplement: Supplementary file 1 — Supplemental Material [file 41393_2024_981_MOESM1_ESM.pdf]

Supplementary Material

Validity and reliability study of a novel surface electromyography sensor using a well-consolidated electromyography system in individuals with cervical spinal cord injury

Chandrasekaran Jayaraman[1] • Chaithanya Krishna Mummidisetty[1] • Arun Jayaraman[1] • Kimberly Pfleeger[2] • Michelle Jacobson[2] • Melissa Ceruolo[3] • Ellora Sen-Gupta[3] • James Caccese[3] • David Chen[1]

[1]Max Näder Center for Rehabilitation Technologies and Outcomes Research, Shirley Ryan AbilityLab, Chicago, IL, USA

[2]AbbVie Inc, North Chicago, IL, USA

[3]Medidata Solutions, a Dassault Systèmes company, New York, NY, USA

Supplementary Table 1. BioStamp nPoint sensor specifications.

**Parameter**	**Value**
Dimensions	7.1 x 3.4 x 0.5 cm (L x W x H maximum)
Weight	8.7 grams
Material	Low durometer silicone
Accelerometer range	± 2-16 g0 ± 10%
Accelerometer bit depth	16
Accelerometer precision	0.6 mg
Accelerometer sample rate	15.625-250 Hz ± 2%
Accelerometer zero-g output	± 60 mg
Gyroscope range	± 250-2000 dps ± 10%
Gyroscope bit depth	16
Gyroscope resolution	0.07 dps
Gyroscope sample rate	15.625-250 Hz ± 2%
Gyroscope zero rate output	± 5 dps
1-lead analog front end range	± 300 mV
1-lead analog front end resolution	10 mV
1-lead analog front end bit depth	16
1-lead analog front end sampling rate	125-1000 Hz ± 2%

dps degrees per second, *g* gravity, g0 standard gravity, *Hz* hertz, *mg* milligravity, *mV* milivolt, *V* volt.

Supplementary Table 2. Delsys system specifications.

Parameter	Value
RF frequency band	2400-2483 MHz (ISM band)
Dimension	27 x 37 x 13 mm
Mass	14 g
Temperature range[a]	5-45 degrees Celsius
EMG signal input range	11 mV / 22 mV rti.
EMG signal bandwidth	20-450 Hz / 10-850 Hz
EMG contact dimensions	5 x 1 mm
Contact material[b]	99.9% silver
Accelerometer range	± 2 g, ± 4 g, ± 8 g, ± 16 g
Accelerometer bandwidth	24 Hz-1473 Hz (configurable in software)
Gyroscope range	± 250 dps, ± 500 dps, ± 1000 dps, ± 2000 dps
Gyroscope bandwidth	24 Hz-360 Hz (configurable in software)
Magnetometer range	± 4900 µT
Magnetometer bandwidth	50 Hz
Inter-sensor delay	< 1 sample period (base station only)
Intra-channel delay	< 1-2 sample period
Analog output range	± 5 V (base station only
Analog output bandwidth (Ch. X.1)	DC-500 Hz (base station only)
Analog output bandwidth (Ch. X.2, X.3, X.4)	DC-50 Hz (base station only)

[a]Exposure beyond these temperature limits may damage the rechargable battery.

[b]Sensor skin contacts are made from pure silver and should not be used if allergic reactions to silver are expected or found to occur.

Ch channel, *DC* direct current, *dps* degrees per second, *EMG* electromyography, *g* gravity, *Hz* hertz, *ISM* Industiral Scientific and Medical, *mm* milimeter, *mV* milivolt, *RF* radio frequency, *rti* referred-to-input, *V* volt, *µT* micro Tesla.

Supplementary Table 3. Differences between Delsys Trigno Wireless EMG and BioStamp nPoint.

Item	Delsys Trigno Wireless EMG	BioStamp nPoint
Amplification	1000 Hz	Default manufacturer setting
Hardware Filter	Analog EMG Sensor Butterworth filter bandwidth: 2 pole high pass corner, 4 pole low pass corner in Hz	Sinc filter: variable decimation rate, third-order, low-pass filter
Sampling Frequency	2000 Hz	1000 Hz
Type of electrode	Bipolar	Bioplar
Inter-electrode distance	1cm	45 mm
Electrode Placement	On muscle belly	On muscle belly

EMG electromyography, *Hz* hertz.

Supplementary Table 4. Anatomical landmarks for electrode placement[11]

Muscle	Anatomical Landmark Frame
Long head biceps brachii	A line between the acromion and the distal insertion of the biceps brachii tendon
Long head of triceps	The line between the angle of the acromion and the medial epicondyle. The sensors were positioned medially inclined with respect to the ALF, on the belly of the muscle.
Rhomboid major	A line between the spinous of the third thoracic vertebrae and the medial border of the scapula. The line was drawn at an angle of 45° to the sagittal plane (T2-T5 spine level).
Extensor carpi radialis longus major	The superficial muscle in the posterior compartment of the forearm.
Extensor carpi radialis bravis	The superficial layer of the posterior compartment of the forearm.

ALF anatomical landmark frame.

Supplementary Table 5. Time domain results for the test-retest reliability correlation between measurements.

Muscle	Time Domain: Trial 1 vs. Trial 2 (Pearson's Correlation Coefficient)							
	Signal-to-Noise Ratio		Peak Amplitude		Burst Duration		Burst Variance	
	BioStamp nPoint	Delsys	BioStamp nPoint	Delsys	BioStamp nPoint	Delsys	BioStamp nPoint	Delsys
Biceps, L	0.81	0.51	0.77	0.74	0.70	0.71	0.81	0.67
Biceps, R	0.78	0.69	0.93	0.95	0.78	0.65	0.91	0.95
Triceps, L	0.62	0.95	0.89	1.00	0.85	0.62	0.94	1.00
Triceps, R	0.56	0.83	0.97	1.00	0.86	0.46	0.98	1.00
Rhomboid, L	0.94	0.77	0.84	0.93	0.70	0.64	0.71	0.94
Rhomboid, R	0.44	0.67	0.83	0.99	0.44	0.61	0.35	1.00
Extensor carpi radialis longus, L	0.89	0.53	0.97	0.91	0.85	0.72	0.98	0.97
Extensor carpi radialis longus, R	0.86	0.76	0.86	0.41	0.88	0.72	0.90	0.34
Extensor carpi radialis brevis, L	0.76	Same as carpi L	0.94	Same as carpi L	0.91	Same as carpi L	0.98	Same as carpi L
Extensor carpi radialis brevis, R	0.91	Same as carpi L	0.98	Same as carpi L	0.79	Same as carpi L	0.98	Same as carpi L

Pearson's correlation: insubstantial (< 0.10), weak (0.10 to <0.29), moderate (0.30 to <0.50), and strong ($\geq$0.50).

BioStamp nPoint BioStamp nPoint electromyography system; *Delsys* Delsys Trigno Wireless electromyography system; *L* Left; *R* Right.

Supplementary Table 6. Frequency domain results for the test-retest reliability correlation between measurements.

	Frequency Domain: Trial 1 vs. Trial 2 (Pearson's Correlation Coefficient)					
	Mean Frequency		Median Frequency		Peak Frequency	
**Muscle**	**BioStamp nPoint**	**Delsys**	**BioStamp nPoint**	**Delsys**	**BioStamp nPoint**	**Delsys**
Biceps, L	0.85	0.87	0.80	0.91	0.49	0.37
Biceps, R	0.88	0.92	0.79	0.91	0.52	0.54
Triceps, L	0.92	0.90	0.86	0.81	0.87	0.99
Triceps, R	0.86	0.67	0.83	0.90	0.81	0.76
Rhomboid, L	0.92	0.77	0.95	0.92	0.92	0.08
Rhomboid, R	0.54	0.92	0.75	0.86	0.63	0.74
Extensor carpi radialis longus, L	0.89	0.73	0.89	0.85	0.88	0.16
Extensor carpi radialis longus, R	0.89	0.74	0.90	0.73	0.40	0.14
Extensor carpi radialis brevis, L	0.77	Same as carpi L	0.80	Same as carpi L	0.86	Same as carpi L
Extensor carpi radialis brevis, R	0.80	Same as carpi	0.80	Same as carpi	0.62	Same as carpi

Pearson's correlation: insubstantial (<0.10), weak (0.10 to <0.29), moderate (0.30 to <0.50), and strong (≥0.50).
BioStamp nPoint BioStamp nPoint electromyography system; *Delsys* Delsys Trigno wireless electromyography system; *L* Left; *R* Right.

Supplementary Table 7. Time domain results for the between-sensor comparisons.

	Time Domain (Spearman's Correlation Coefficient)			
	Signal-to-Noise Ratio	Peak Amplitude	Burst Duration	Burst Variance
**Muscle**	**BioStamp nPoint vs. Delsys**	**BioStamp nPoint vs. Delsys**	**BioStamp nPoint vs. Delsys**	**BioStamp nPoint vs. Delsys**
Biceps, L	0.68*	0.48*	0.75*	0.43*
Biceps, R	0.87	0.82*	0.90*	0.78*
Triceps, L	0.87*	0.89*	0.82*	0.84*
Triceps, R	0.68	0.93*	0.81*	0.89*
Rhomboid, L	0.40	0.80*	0.71*	0.80*
Rhomboid, R	0.61	0.80	0.55	0.80
Extensor carpi radialis longus, L	0.04*	0.07	0.89	0.003*
Extensor carpi radialis longus, R	0.16	0.05	0.75	0.13

Spearman's correlation: insubstantial (<.10), weak (.10 to <0.29), moderate (0.30 to <0.50), and strong ($\geq$0.50).

Statistical significance comparing the signal mean values was determined using the non-parametric Wilcoxon signed-rank test.

*P < 0.05.

Delsys Delsys Trigno^{TM} wireless electromyography system; *L* Left; *BioStamp nPoint* BioStamp nPoint electromyography system; *R* Right.

Supplementary Table 8. Frequency results for the between-sensor comparisons.

	Frequency Domain (Spearman's Correlation Coefficient)		
	**Mean Frequency**	**Median Frequency**	**Peak Frequency**
**Muscle**	**BioStamp nPoint vs. Delsys**	**BioStamp nPoint vs. Delsys**	**BioStamp nPoint vs. Delsys**
Biceps, L	0.28*	0.42*	0.10
Biceps, R	0.47*	0.57*	0.26
Triceps, L	0.20*	0.25*	0.29
Triceps, R	0.51*	0.85*	−0.16
Rhomboid, L	0.62*	0.85*	0.60
Rhomboid, R	0.47	0.68	0.74
Extensor carpi radialis longus, L	0.16	0.13	0.05
Extensor carpi radialis longus, R	0.42	0.46	0.57

Spearman's correlation: insubstantial (<.10), weak (.10 to <0.29), moderate (0.30 to 0.49), and strong (≥0.50).

Statistical significance comparing the signal mean values was determined using the non-parametric Wilcoxon signed-rank test.

**P* < 0.05.

BioStamp nPoint BioStamp nPoint electromyography system; *Delsys* Delsys Trigno wireless electromyography system; *L* Left; *R* Right.

Supplementary Table 9.

Optimal muscles for sEMG measurements using BioStamp nPoint[a] electromyography system.

**Muscle**	**Right Side Signal-to-Noise Ratio/Biosensor Placement**	**Left side Signal-to-Noise Ratio/Biosensor Placement**
Biceps	22.1 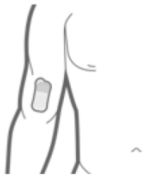	19.6 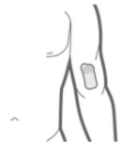
Triceps	12.5 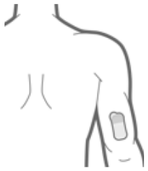	11.0 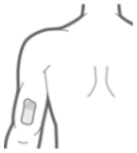
Extensor carpi radialis longus	11.5 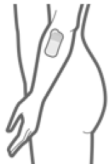	12.5 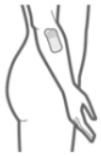
Rhomboid	8.0 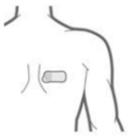	7.1 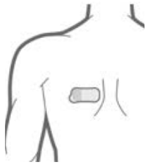

[a]Data are ranked based on signal-to-noise ratio.

sEMG Surface electromyography.
